# Screening of intact yeasts and cell extracts to reduce Scrapie prions during biotransformation of food waste

**DOI:** 10.1186/s13028-018-0363-y

**Published:** 2018-02-08

**Authors:** David Huyben, Sofia Boqvist, Volkmar Passoth, Lena Renström, Ulrika Allard Bengtsson, Olivier Andréoletti, Anders Kiessling, Torbjörn Lundh, Ivar Vågsholm

**Affiliations:** 1 Department of Animal Nutrition and Management, Swedish University of Agricultural Sciences, 75007 Uppsala, Sweden; 2 Department of Biomedical Sciences and Veterinary Health, Swedish University of Agricultural Sciences, 75007 Uppsala, Sweden; 3 Department of Molecular Sciences, Swedish University of Agricultural Sciences, 75007 Uppsala, Sweden; 4 Department of Microbiology, National Veterinary Institute, 75189 Uppsala, Sweden; 5 Interactions Hôte Agent Pathogéne, École Nationale Vétérinaire de Toulouse, 31300 Toulouse, France

**Keywords:** Food waste, Prions, Protease, Yeast

## Abstract

Yeasts can be used to convert organic food wastes to protein-rich animal feed in order to recapture nutrients. However, the reuse of animal-derived waste poses a risk for the transmission of infectious prions that can cause neurodegeneration and fatality in humans and animals. The aim of this study was to investigate the ability of yeasts to reduce prion activity during the biotransformation of waste substrates—thereby becoming a biosafety hurdle in such a circular food system. During pre-screening, 30 yeast isolates were spiked with Classical Scrapie prions and incubated for 72 h in casein substrate, as a waste substitute. Based on reduced Scrapie seeding activity, waste biotransformation and protease activities, intact cells and cell extracts of 10 yeasts were further tested. Prion analysis showed that five yeast species reduced Scrapie seeding activity by approximately 1 log10 or 90%. *Cryptococcus laurentii* showed the most potential to reduce prion activity since both intact and extracted cells reduced Scrapie by 1 log10 and achieved the highest protease activity. These results show that select forms of yeast can act as a prion hurdle during the biotransformation of waste. However, the limited ability of yeasts to reduce prion activity warrants caution as a sole barrier to transmission as higher log reductions are needed before using waste-cultured yeast in circular food systems.

## Findings

Prions are misfolded proteins that can cause transmissible spongiform encephalopathies (TSEs) that result in fatal neurodegeneration [1, 2]. The infectious form occurs when the cellular membrane bound prion protein (PrP^c^) is converted into a partially protease-resistant pathologic form (PrP^Sc^) [3]. Infectious prions can cause diseases such as Creutzfeldt–Jakob Disease (CJD) in humans, bovine spongiform encephalopathy (BSE) in cattle, Scrapie in sheep and goats, and chronic wasting disease (CWD) in deer [2, 4]. Human dietary exposure to BSE prions resulted in the emergence of a new form of human TSE (variant CJD) and has led to feeding bans of specific risk material, such as brain and spinal cord, to other animals [5, 6]. However, prion transmission still occurs in livestock farms, such as prevalence of Scrapie in Greek sheep [6], and in wild populations, such as the recent CWD outbreak in Norwegian reindeer [7], that poses a substantial risk to the safety of food and public health.

There is increasing pressure to produce more food with fewer resources to feed the growing human population in spite of the depletion of phosphorus, limited arable land and negative effects of climate change. One promising solution to this challenge is to use microorganisms that convert organic waste into high quality protein and lipid sources for animal feeds and even human food [8], thereby recapturing nutrients in a circular food production system. However, organic waste may possess infectious prions from animal trimmings, thus recapturing nutrients may also transmit prions.

Recent studies have shown that microorganisms, such as bacteria and fungi, derived from sediment, soil and lichens can degrade PrP^Sc^ using their proteolytic enzymes [9–11]. Species of yeast have been shown to convert organic waste to high-quality animal feed [12, 13] and exhibit high protease activity [14]. These combined properties may represent a solution to tackle the prion-associated risk during recycling of animal-derived waste while producing feed. In this study we characterized the impact of intact yeast cells and yeast cell extracts on the activity of Classical Scrapie prions during the biotransformation of casein as a waste substitute.

Yeasts strains were obtained from the Department of Molecular Science, SLU (Uppsala, Sweden) and pre-cultured from freezer stocks on media of yeast peptone dextrose (YPD) [15]. After pre-culture, yeasts were cultured in YPD broth at 25 °C in a shaker at 120 rpm for 24 h. Yeasts were centrifuged at 3000*g*, washed twice with phosphate buffer saline and diluted to an OD_600nm_ of 2.0. Separately, yeast extracts were prepared by centrifugation at 5000*g*, 0.2 μm filter sterilization of supernatant and 20-fold concentration with 10 kDa centrifugal filters (Merck, Solna, Sweden) [16]. Total protease activity was measured by incubating intact and extracted yeasts with casein labelled with fluorescein isothiocyanate for 1 h at 37 °C and measuring fluorescence, according to the manufacturer (Sigma-Aldrich, Stockholm, Sweden).

The Classical Scrapie isolate was derived from experimentally VRQ/VRQ affected sheep (PG127) and end titrated from inoculated tg338 mice at the terminal stage (10^7.6^ ID50/g) [17, 18]. The brain homogenate was serially diluted with glucose to a 10% solution.

Initial screening of yeast with Scrapie was performed at the Swedish National Veterinary Institute (SVA; Uppsala, Sweden) where risk group 3** agents are handled according to Swedish legislation. Aliquots of 100 μL of each yeast isolate (Table 1) were incubated with 800 μL of casein substrate (10 g/L tryptone casein, 10 g/L dextrose, 1 g/L yeast nitrogen base and 0.66 g/L ammonium sulphate) and spiked with 100 μL of Scrapie (final dilution of 10^6.6^ ID50/g) in a permeable deep-well microflask (Applikon, Foster City, CA, USA). Positive and negative controls (presence/absence of yeast and/or Scrapie) at 0 and 72 h of incubation were included. Microflasks were incubated at 20 °C for 72 h in a shaker at 300 rpm to mimic yeast production conditions. Each reaction was stopped by adding yeast protease inhibitor (Roche, Basel, Switzerland) and stored at − 80 °C.

**Table 1:** Ability of intact yeast isolates to grow on waste, produce proteases and reduce Scrapie activity

Yeast species	Reference strain	SLU strain	Waste substrate^a^	Protease actviity^b^	Scrapie reduction^c^	Highest detection^d^
Ascomycota phyla						
*Aureobasidium pullulans*	NA	J126	NA	NA	2/2	10^−5.5^
*Blastobotrys adeninivorans*	CBS 8244	J562	Plant, dairy	Low	1/2	10^−5.0^
*Cyberlindnera jadinii*	CBS 621	J556	Plant, dairy	High	1/2	10^−5.0^
*Debaryomyces hansenii*	CBS 1962	J136	Dairy	Low	1/2	10^−6.0^
*Debaryomyces hansenii*	CBS 6958	J187	Dairy	Low	0/2	10^−6.0^
*Debaryomyces hansenii*	NRRL 7268	J345	Dairy	Low	1/2	10^−6.0^
*Diutina catenulata*	NA	J598	Plant, dairy	High	2/2	10^−5.0^
*Kluyveromyces lactis*	CBS 2359	J469	Plant, dairy	NA	0/2	10^−6.0^
*Kluyveromyces marxianus*	CBS 6556	J137	Dairy	High	1/2	10^−6.0^
*Kluyveromyces marxianus*	CBS 1089	J186	Plant, dairy	High	2/2	10^−5.0^
*Kluyveromyces marxianus*	CBS 1555	J367	Dairy	High	0/2	10^−6.0^
*Ogataea polymorpha*	CBS 4732	J549	Plant, dairy	Low	1/2	10^−5.5^
*Pichia kudriavzevii*	CBS 2062	J550	Plant, dairy	NA	1/2	10^−5.5^
*Saccharomyces cerevisiae*	CBS 2978	J122	Plant, seafood	Low	0/2	10^−6.0^
*Saccharomyces cerevisiae*	NA	J545	Plant, seafood	Low	1/2	10^−5.5^
*Saccharomyces cerevisiae*	NA	J546	Plant, seafood	Low	1/2	10^−5.0^
*Scheffersomyces stipitis*	CBS 5774	J563	Plant, dairy	Low	0/2	10^−6.0^
*Torulaspora delbrueckii*	NA	J352	Plant, dairy	NA	1/2	10^−5.5^
*Wickerhamomyces anomalus*	CBS 100487	J121	Plant, dairy	Low	0/2	10^−6.0^
*Wickerhamomyces anomalus*	CBS 1947	J379	Plant, dairy	Low	0/2	10^−7.0^
*Wickerhamomyces anomalus*	CBS 100487	J475	Plant, dairy	Low	0/2	10^−6.0^
*Yarrowia lipolytica*	CBS 6114	J134	Plant, dairy, seafood	High	1/2	10^−5.5^
Basidiomycota phyla						
*Cryptococcus laurentii*	CBS 6473	J463	NA	Low	2/2	10^−5.5^
*Holtermanniella takashimae*	CBS 11174	J596	Plant	NA	1/2	10^−5.0^
*Naganishia cerealis*	NA	J595	Plant, dairy	NA	2/2	10^−4.5^
*Phaffia rhodozyma*	NA	J552	Plant, dairy	NA	0/2	10^−7.0^
*Rhodotorula glutinis*	NA	J195	Plant, dairy	High	1/2	10^−5.5^
*Sporidiobolus pararoseus*	CBS 4216	J466	Plant, dairy	High	0/2	10^−6.0^
*Sporidiobolus roseus*	NA	J104	NA	NA	0/2	10^−6.0^
*Sporidiobolus salmonicolor*	CBS 490	J360	NA	NA	1/2	10^−5.5^

*CBS* Central Bureau for Fungus Cultures (Utrecht, Netherlands); *NRRL* Northern Regional Research Laboratory (Peoria, IL, USA); *NA* not available; *SLU* Swedish University of Agricultural Sciences (Uppsala, Sweden)

^a^Food grade yeasts that have been cultured on these waste substrates [12, 13]

^b^High indicates that > 50% of isolates were reported to produce protease and low indicates < 50% [14]

^c^Number of positive tests that showed 1–2 log10 reduction in Scrapie activity after incubation for 72 h

^d^Mean dilution of highest positive detection of Scrapie after immunoblotting

Protein misfolding cyclic amplification (PMCA) was used to measure the level of prion seeding activity by amplifying minute amounts of PrP^Sc^ at ENVT/INRA (Toulouse, France) [19]. The reaction product was serially diluted 1:10 and used as a seed in PMCA where 5 μL was added to 45 μL of 10% tg338 brain homogenate and amplified using two rounds of 96 cycles of 10 s sonication with 14 min and 50 s of rest at 38.5 °C (QSonica, Newtown, CT, USA) [20]. Presence of PrP^Sc^ was detected by Dot blotting using a microfiltration apparatus according to the manufacturer (Bio-Rad, Hercules, CA, USA) and immunoblotting with Sha31 anti PrP monoclonal antibody according to [20]. Scrapie presence in each sample was compared to the positive-Scrapie control at 72 h to account for reductions in seeding activity from 0 h due to incubation with the yeast substrate.

Initial screening indicated that 19 out of 30 intact yeast isolates resulted in 1–2 log10 reduction of the Scrapie seeding activity (Table 1). Among these isolates, 10 were selected for further testing based on different criteria. Some strains, like *Diutina catenulata* (J598) and *Kluyveromyces marxianus* (CBS 1089) were selected due to their reported ability to culture on plant and dairy wastes, their high protease activity and their ability to reduce Scrapie activity. Others like *Kluyveromyces lactis* (CBS 2359), *Phaffia rhodozyma* (J552) and *Scheffersomyces stipites* (CBS 5774) were excluded because of their low reported protease activity and their lack of Scrapie reduction.

The 1:10 dosage of Scrapie spiked in the yeast cultures was observed to inhibit yeast growth, thus the next experiment used a 1:100 dilution. For intact yeast, incubation was repeated as previously described, except 890 μL of casein substrate was spiked with 10 μL of Scrapie (final dilution of 10^5.6^ ID50/g) and incubated with 100 μL of intact yeast. For yeast extracts, 135 μL was incubated with 15 μL of Scrapie for 24 h at 30 °C in a micro-titre plate.

These new tests indicated that that the processing of the substrate by five out of the 10 intact and/or cell extracts resulted in a 90% decrease (approximatively 1 log10) of the PG127 Scrapie seeding activity (Table 2). Both the intact cells and extracts of *Cryptococcus laurentii* reduced Scrapie activity. Intact forms of *D. Catenulata* and *Wickerhamomyces anomalus* and cell extracts of *Blastobotrys adeninivorans* and *Debaryomyces hansenii* also reduced Scrapie seeding activity. Interestingly, the three yeast proteases that reduced Scrapie also had the highest level of protease activity, which was especially high for *Cr. Laurentii.* In contrast, protease activity was lowest for intact yeast species of *D. Catenulata* and *W. Anomalus*, while they were still able to reduce Scrapie activity.

**Table 2:** Reduction of Scrapie activity by intact and extracted yeasts and their corresponding protease activity

Yeast species	Scrapie reduction^a^ and highest detection^b^		Protease activity^c^			
	Intact yeast	Extracted yeast	Intact yeast		Extracted yeast	
*Aureobasidium pullulans*	0/4 10^−4.1^	0/2 10^−3.3^	18.6 (4.9)		13.6 (16.7)	
*Blastobotrys adeninivorans*	0/4 10^−3.9^	1/2 10^−3.0^	16.2 (3.8)		144.7 (60.1)	
*Cryptococcus laurentii*	3/4 10^−3.3^	2/2 10^−2.8^	33.4 (48.9)		286.5 (6.6)	
*Debaryomyces hansenii*	0/4 10^−4.8^	2/2 10^−3.0^	42.3 (11.0)		61.7 (41.8)	
*Diutina catenulata*	2/4 10^−3.4^	0/2 10^−3.8^	16.7 (2.8)		22.5 (1.7)	
*Holtermanniella takashimae*	0/4 10^−4.1^	0/2 10^−3.5^	19.8 (0.6)		19.7 (0.1)	
*Kluyveromyces marxianus*	0/4 10^−3.6^	0/2 10^−3.8^	39.8 (46.3)		48.7 (1.1)	
*Naganishia cerealis*	0/4 10^−4.3^	0/2 10^−4.0^	7.1 (2.1)		43.4 (63.0)	
*Saccharomyces cerevisiae*	0/4 10^−3.5^	0/2 10^−4.0^	18.6 (2.3)		23.6 (2.6)	
*Wickerhamomyces anomalus*	3/4 10^−3.1^	0/2 10^−3.5^	12.9 (3.1)		21.5 (0.1)	

^a^Number of positive tests that showed 0.5–1.0 log10 reduction in Scrapie after incubation compared with a control

^b^Mean dilution of highest positive detection of Scrapie after immunoblotting

^c^Analysed by the fluorescence detection of trypsin (ng) from casein substrate after 60 min incubation at 37 °C

More than 5 log10 reduction was needed to safely eliminate prions in this study, thus 1 log10 reduction was insufficient. Moreover, even higher inactivation is needed depending on the source material and its infectivity [21, 22]. Therefore, these findings indicate the potential of yeast biotransformation as one hurdle, but the limited reduction indicates additional hurdles, such as heat treatment, are needed before using waste-cultured yeast as animal feed or food [2, 23].

This study is the first attempt to determine the ability of yeasts to reduce prion activity. However, there are several ways this approach could be improved. First, the number of yeast isolates tested could be expanded and even filamentous fungi should be included since they too have been used in animal feed. Second, the period of yeast–Scrapie incubation (i.e. 72 h) could be increased, such as 8 days used previously with soil microbes [11], to increase the likelihood of Scrapie reduction. Third, the pH and temperature could be increased, such as pH 10 and > 30 °C used previously with bacterial proteases [9, 24, 25], although these conditions may impact yeast growth and jeopardise their use as animal-grade feed. Fourth, different techniques could be used to produce cell extracts, such as acetone extraction as described previously [10], that may improve prion reduction.

Scrapie reduction by two intact yeasts with low protease activity was unexpected (Table 2) since prion degradation has been shown to be mediated by serine proteases [10]. In a similar study, 199 food-borne bacterial isolates were screened for Scrapie reduction and six were positive, while one isolate had low protease activity [16]. These findings suggest that total protease activity is not the only defining aspect that enables yeasts to degrade prions. In addition, the aforementioned study noted the bacterial isolates represent four different genera and suggested that their enzymes may share specific properties that allow them to effectively degrade Scrapie [16]. In comparison, four out of five yeast isolates in the present study belong to the phylum Ascomycetes, order Saccharomycetales, while the most promising isolate, *Cr. Laurentii,* belongs to the phylum Basidiomycetes, order Tremellales. More research is needed to compare the enzymatic properties of different groups of yeast and determine the underlying mechanism that enables yeast to degrade Scrapie prions.

In conclusion, these results indicate that some yeast species, both as intact cells and cell extracts, have the potential to reduce the transmission of prions while converting organic waste to high-quality animal feed, thereby becoming a prion hurdle. This study is an important first step, although additional hurdles are required to prevent the transmission of prions into circular food systems.

## Abbreviations

BSE: bovine spongiform encephalopathy
CJD: Creutzfeldt–Jakob disease
CWD: chronic wasting disease
ENVT: National Veterinary School of Toulouse/École nationale vétérinaire de Toulouse
ID50: infectious dose 50
INRA: French Agronomic Research Institute/L’Institut national de la recherche agronomique
PMCA: protein misfolded cyclic amplification
SLU: Swedish University of Agricultural Sciences/Sveriges Lantbruksuniversitet
SVA: Swedish National Veterinary Institute/Statens Veterinärmedicinska Anstalt
TSE: transmissible spongiform encephalopathy
YPD: yeast peptone dextrose
